# Informing a governance model for integration of community pharmacists and family physicians and nurse practitioner-led practices and teams within Ontario Health Teams: A protocol

**DOI:** 10.1371/journal.pone.0325270

**Published:** 2025-06-17

**Authors:** Monica Aggarwal, Lisa Dolovich, Yazid Al Hamarneh, Kristina M. Kokorelias, Reed Beall, Ross Upshur

**Affiliations:** 1 Dalla Lana School of Public Health, University of Toronto, Toronto, Ontario, Canada; 2 Department of Family and Community Medicine, University of Toronto, Toronto, Ontario, Canada; 3 Leslie Dan Faculty of Pharmacy, University of Toronto, Toronto, Ontario, Canada; 4 Department of Pharmacology, University of Alberta, Edmonton, Alberta, Canada; 5 Section of Geriatrics, Department of Medicine, Sinai Health and University Health Network, Toronto, Ontario, Canada; 6 Department of Occupational Sciences and Occupational Therapy, University of Toronto, Toronto, Ontario, Canada; 7 Rehabilitation Sciences Institute, University of Toronto, Toronto, Ontario, Canada; 8 Department of Community and Health Sciences, University of Calgary, Calgary, Alberta, Canada; 9 O’Brien Institute for Public Health, University of Calgary, Calgary, Alberta, Canada; Endeavour College of Natural Health, AUSTRALIA

## Abstract

**Background:**

The expansion of the scope of practice for community pharmacists has the potential to improve timely access to primary care. However, overlapping scopes of practice with family physicians (FPs) and nurse practitioners (NPs) have the potential for duplication of services or fragmented care. To optimize the benefits of this change, clear integrated governance mechanisms are needed to define roles, responsibilities, and accountabilities between providers. To date, there is no research on integrated governance models for independent primary care provider organizations.

**Objective:**

To develop a governance model that will enable better integration of community pharmacists with FPs and NPs within Ontario Health Teams.

**Methods:**

A multi-method study design will be used. A literature review will be conducted to identify innovative programs and policies between community pharmacies, FPs, and NPs. Then, relevant stakeholders will be identified and engaged in key informant interviews to exchange knowledge, identify priorities, and co-create policy and governance strategies.

**Results:**

The scoping review will identify existing models, gaps, and governance approaches that support or hinder integration. Interviews will explore stakeholder experiences related to integrated governance. A conceptual integrated governance model will be developed based on these findings.

**Discussion:**

This initiative aligns with the broader mission of improving healthcare services and outcomes in Ontario by establishing a governance model that enables better integration between community pharmacists, FPs, and NPs. This research will support policy and practice innovations to strengthen integrated delivery in Ontario and beyond.

## Introduction

Pharmacists play an integral role in the primary care (PC) system. We define PC “as an inclusive term to cover the spectrum of first contact healthcare models from those whose focus is comprehensive, person-centred care, sustained over time, to those that also incorporate health promotion towards community development and intersectoral action to address the social determinants of health” [1]. Pharmacists deliver PC services [2–4] and frequently see patients in the community, more often than other healthcare providers [5]. Pharmacists are trained to provide a wide range of services, including: prescribing medications, ordering lab tests, providing injections for vaccines and other injectable products, delivering patient education on lifestyle and drug usage, overseeing the dispensing of medications, monitoring actual or potential drug effectiveness, harms, or interactions, and offering expertise on pharmaceuticals’ proper use and effects [6]. Pharmacists have varying scopes across Canadian jurisdictions and work in various settings, including community, hospital, and as part of interdisciplinary PC teams [7]. Pharmacists, in any of the aforementioned settings, work collaboratively with other professionals and have demonstrated to improve patient care and outcomes [7].

As of January 1, 2023, the Ontario government granted pharmacists prescribing authority for 13 minor (common) ailments [8]. On October 1, 2023, pharmacists gained prescribing authority for six additional conditions, bringing the total to 19 [9]. These legislative changes align with developments in other provinces (Newfoundland, New Brunswick, and Alberta) [9] and countries (New Zealand, the United Kingdom, and the United States) [10] that have expanded the scope of practice for pharmacists autonomously or in collaboration with other healthcare providers to prescribe and renew medications. This expansion is reshaping PC by enabling pharmacists to deliver services traditionally provided by family physicians (FPs) or nurse practitioners (NPs) (e.g., prescribing medications, adjusting dosages, administering vaccinations, managing chronic conditions [e.g., diabetes, hypertension]) [11,12]. In underserved areas, pharmacists’ expanded prescribing authority could alleviate gaps in healthcare access, offering timely care for patients who might otherwise face long wait times or travel significant distances [7,12]. However, in more well-served areas with strong provider networks, the impact may be more modest and could potentially divert attention and resources from strengthening PC services in underserved regions.

Nevertheless, these reforms represent a promising step toward broader pharmacist integration in PC [13]. Several models now embed pharmacists in physician or NP-led interprofessional PC teams [14–19]. Meanwhile, community pharmacists are working in independently owned pharmacies, banner, chain pharmacy corporations and other types of pharmacies [20], where patients can be assessed and treated for injuries and common ailments such as urinary tract infections and pink eye [21] and receive care for diabetes, hypertension, and asthma with or without needing an appointment [21]. In some models, the NP is part of the team, while in others, pharmacists provide PC services within their autonomous community pharmacy setting [22,23]. For example, in Nova Scotia, pharmacists in Community Pharmacy Primary Care Clinics are responsible for addressing 31 minor ailments and prescribing and monitoring medications for common chronic conditions such as diabetes [24].

The integration of community pharmacists into the PC system can optimize overall healthcare delivery and patient health in the community [25]. Integration can be defined as “a coherent set of methods and models on the funding, administrative, organizational, service delivery and clinical levels designed to create connectivity, alignment and collaboration within and between the cure and care sectors” [26] Since community pharmacists, FPs, and NPs increasingly share scopes of practice—and sometimes the same patients—integration between providers and organizations will be crucial to avoid fragmented care. Effective integration can improve processes (e.g., appropriate medication use, better hypertension control, increased medication consultations, enhanced prescribing practices) and outcomes (reduced healthcare utilization and medication costs, and better management of chronic conditions) [7]. The outcome of successful integration processes within the clinical setting can be termed integrated care, [26] which is operationalized in this paper as different health services embedded in a single unified system with shared governance and processes [27–29]. Integrated care models exist on a spectrum, ranging from simple communication between providers to fully integrated interprofessional teams [30].

Establishing effective governance models to foster integrated care is essential for defining the roles and authority of key stakeholders (e.g., FPs, NPs, PC teams, or organizations, and community pharmacists), establishing mechanisms for resolving conflicts, making strategic decisions, maintaining compliance with legal and ethical standards; and ensuring there is accountability, transparency, and effective use of resources [31]. Governance is defined as a combination of structures, policies, and processes encompassing legal agreements and addressing shared and sole risks where applicable [32] to ensure that a designated governance body delivers value to shareholders [33]. Decision-making, resource allocation, and responsibilities can be shared, emphasizing inclusivity, cooperation, and consensus-based decisions. Research shows that well-structured governance mechanisms can enhance role clarity, trust, communication, and problem-solving and minimize conflicts, ultimately leading to more successful integrations with outcomes (e.g., improved service delivery, innovation, and sustained relationships between the involved organizations) [34]. However, challenges may arise from power imbalances among stakeholders, conflicting interests, and differing organizational cultures, which can impede effective integration [34,35].

Successful integration is defined as the development of a well-functioning and integrated working partnership between community pharmacists and FPs and NPs [36–39]. Although there is a substantial amount of literature on collaboration between FPs and pharmacists in the context of those formally affiliated/connected with a PC practice or team [40], there is less known about governance models [41] that formally establish the roles and responsibilities within a health system for integrated care delivered by community pharmacy teams (e.g., teams that encompass pharmacists, pharmacy technicians and pharmacy assistants) who are working in independent, often/mainly privately owned organizations and FP and NP-led practices and teams. The development of governance mechanisms for effective integration between community pharmacists and FP and NP-led practices or teams within the health care system will be pivotal to advancing coordinated, effective, accessible, and quality care to Ontarians.

This proposal aims to generate evidence to inform the development of a governance model to enable better integration between community pharmacists and FP and NP-led practices or teams within Ontario Health Teams (OHTs). OHTs are collaborative networks of healthcare providers and organizations working together to deliver a continuum of care to a defined population, from primary, acute, long-term, and home care to social and community services. OHTs are designed to streamline patient transitions between providers, improve care integration, and share resources under a single accountability framework [42,43]. OHTs provide an opportune setting for implementing the proposed integrated governance model, as they are designed to enhance collaboration across healthcare sectors and facilitate interdisciplinary coordination.

## Methods

### Design

A multi-methods study design, informed by other PC studies, is proposed for this study. This study will be theoretically informed by two frameworks: the RACI Matrix (Responsible, Accountable, Consulted, Informed) and the Nine Pillars of Integrated Care. The RACI matrix is a responsibility assignment tool that clarifies roles in a task or project, helping to streamline decision-making by defining who is responsible, who has authority, and who should be consulted or informed [44,45].

The International Foundation for Integrated Care (IFIC) developed the Nine Pillars of Integrated Care as foundational principles for enhancing population health and well-being through collaborative systems [46]. These include: (1) establishing shared values and vision; (2) adopting a population health approach that addresses root causes and social determinants; (3) engaging people as active partners in their health and care; (4) fostering resilient communities and new alliances; (5) developing workforce capacity and capability; (6) implementing system-wide governance and leadership; (7) leveraging digital solutions; (8) aligning payment systems; and (9) ensuring transparency of progress, results, and impact [46]. Together, these principles underscore the significance of person-centred, collaborative approaches that transcend traditional healthcare boundaries, emphasizing community empowerment, digital innovation, and value-based care models [46].

**Fig 1:**
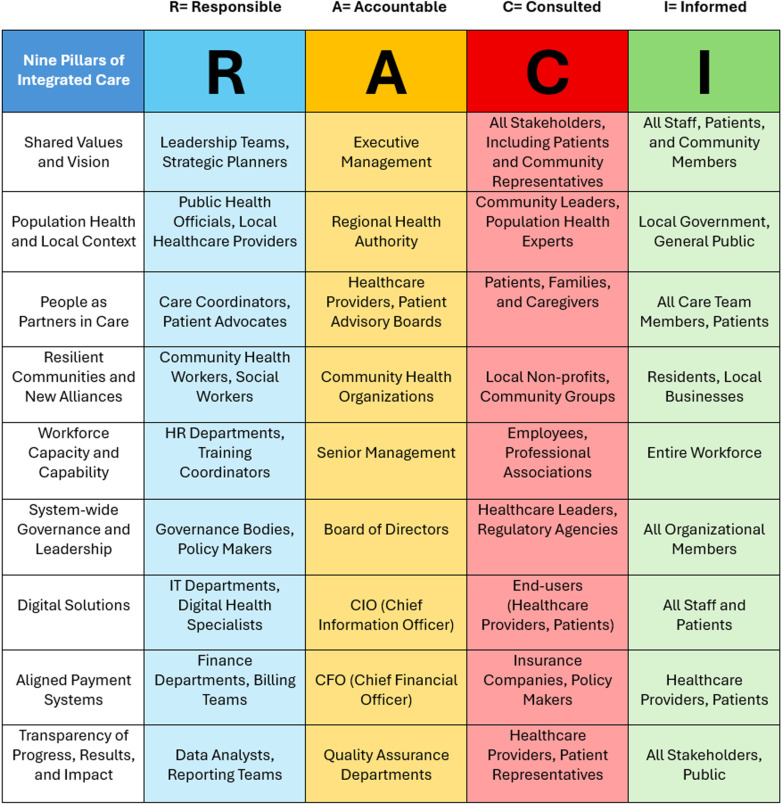
Nine Pillars of Integrated Care mapped to RACI matrix categories.

The RACI matrix and the Nine Pillars of Integrated Care share a common focus on enhancing clarity, accountability, and collaboration in healthcare management. To illustrate this alignment, we created a figure (Fig 1) mapping the components of the RACI matrix—defining roles as Responsible, Accountable, Consulted, and Informed—to the Pillars’ emphasis on system-wide governance and leadership (Pillar 6), which calls for clear delineation of roles and responsibilities within integrated care systems [44,46]. This figure was developed based on our understanding of the core principles and applications of both frameworks [44,46], informed by existing literature on the different stakeholders involved in healthcare decision-making [47–49] (i.e., A = Accountability aligns the “System-wide Governance and Leadership” pillar with the Board of Directors as “Accountable” and healthcare leaders as “Consulted” to reflect established governance practices in healthcare organizations) [50]. This figure is, therefore, grounded in the researchers’ knowledge of the literature. It will be further validated and refined through this research to ensure its accuracy and utility.

To meet our objectives, we will conduct a structured literature review on programs between community pharmacy practices or teams (pharmacists, which may also include pharmacy technicians or pharmacy assistants) and FP and NP-led practices and teams. Subsequently, interviews with key stakeholders from these sectors will guide governance strategies for integrated models, focusing on roles, responsibilities, accountabilities, and legal frameworks.

### Phase 1: Literature review

We will conduct a structured literature review informed by the Joanna Briggs Institute [51] methods to identify and analyze best practice programs or innovations involving community pharmacists and FPs and NPs. This review will follow the PRISMA extension for scoping reviews (PRISMA-ScR) guidelines to ensure transparency and rigour [52] (see S1 Fig). We will systematically search multiple databases, including Medline, PubMed, Embase, CINAHL, and PsycINFO, using a combination of relevant keywords and Medical Subject Headings (MeSH) terms. The search strategy will be designed to capture articles, reports, guidelines, and policy documents published within the last decade (from 2008 onwards) (See S2 Fig). We will include studies and documents on initiatives between community pharmacists and FP and NP-led practices and teams. Articles will be excluded if they do not meet these criteria or are not available in English. The screening will occur at two levels (level 1- title and abstract; level 2- full text) through the engagement of a graduate student and member of the research team independently.

Information will be extracted from the selected studies, including governance structures, composition of stakeholders, processes, challenges, enablers, and outcomes. Using elements of the RACI framework, we will analyze the literature to identify: *Who is Responsible? Who is Accountable? Who needs to be Consulted for expert insights? Who should be informed about the progress and findings?* The Nine Pillars of Integrated Care will be used as a framework to evaluate and categorize the presence or absence of the nine key elements of initiatives between community pharmacy and FP and NP-led practices and teams [46]. We will also examine barriers, facilitators, and lessons learned [53,54]. The review will examine the outcomes achieved by programs or innovations in relation to the quintuple aim (i.e., improving population health, enhancing patient experience, reducing costs, supporting care team well-being, and advancing health equity) [55], such as improvements in patient health outcomes, healthcare quality, access to services, and cost-effectiveness.. Data will be systematically organized and summarized in an EndNote database [56]. The quality of included studies will be assessed using appropriate appraisal tools (e.g., the Cochrane Risk of Bias tool for intervention studies [57] and the Joanna Briggs Institute Critical Appraisal Checklist for other study types [58]). This assessment guides evidence weighting and model selection. Besides academic sources, we will target grey literature in Australia, New Zealand, and the United Kingdom (UK), seeking unpublished reports, policies, and guidelines to uncover nuanced insights from established governance models. Examining literature from Australia, New Zealand, and the UK on integrated models is justified due to the shared similarities in their healthcare systems, including integrated PC models and patient responsibilities of pharmacy practice [59]. These countries also serve as leading examples in fostering integrated approaches between FP and NP practices and pharmacies, offering diverse perspectives and best practices applicable to global healthcare settings. This approach enriches our analysis with real-world examples and innovative strategies. After reviewing the literature, we will reach out to leaders of these innovations in the respective countries; if not found, we will proceed to interview Canadian stakeholders.

### Phase 2: Key informant interviews

We will utilize a qualitative descriptive approach [60] to: (a) exchange knowledge about current and desired strategies for a governance model that facilitates integration between community pharmacists and FPs and NPs; (b) identify key priorities to meet the healthcare needs of Ontarians through an integrated PC model; (c) co-create policy and governance components and priorities (e.g., contracts, accountabilities, laws and responsibilities) aligned with the RACI and The Nine Pillars of Integrated Care to support a comprehensive governance model for integrated PC service delivery that includes community pharmacy in Ontario; and (d) identify future lines of research inquiry to support practice and policy to support integrated models of PC within the context of the Ontario health care system. Qualitative descriptive approaches are beneficial when recommendations related to policy and service are the product of the research [60]. This approach leads to more in-depth and rich data than consultative exercises because it engages stakeholders in qualitative descriptive exploration, fostering nuanced understanding and comprehensive insights into the complexities of integrated PC governance. This is crucial to ensure that policy and practice decisions are informed by a deep understanding of stakeholder perspectives, enabling the development of effective, sustainable, and patient-centered healthcare strategies for Ontarians.

We will engage key informants from FPs, NPs, community pharmacists, policymakers, and others impacted by identified innovations. In the absence of such findings, consultations with professional associations, policymakers, and healthcare leaders will guide informing a future governance model. Healthcare leaders, within this context, encompass executives, administrators, managers, or individuals holding influential roles and involved in guiding strategic decisions and policy implementations of the identified innovation. We will use a purposive and snowball sampling strategy to ensure diverse representation of groups. These interviewees are likely to possess extensive expertise and firsthand experience in the healthcare landscape, enabling them to provide valuable insights and perspectives crucial for informing and collaboratively developing governance models tailored to the needs of different communities. To ensure an equitable balance of perspectives, our purposive sampling strategy will oversample participants from groups that may have less formal decision-making power, such as community pharmacists and patients. This approach helps mitigate the risk of dominant voices—such as physicians or healthcare executives—overshadowing others in analyses [61]. We will aim to interview at least 30 key informants or until data saturation is achieved, which is in line with recommendations for qualitative research [62]. We anticipate that 30 interviews will allow for data saturation, although more or fewer interviews may be needed [63]. The interviews will be scheduled based on the convenience of participants. We will use semi-structured interview guides tailored to the role and perspective of each informant group. Tailored semi-structured interview guides will explore the various elements of integration, governance, roles, contractual aspects, and accountabilities, gathering insights for governance design. During our interviews, we will remain mindful of the potential implications of equity, diversity, inclusion, accessibility and anti-racism for integrated governance and be open to discussing how these factors may influence the development and implementation of our proposed models. This approach ensures that our governance strategies are inclusive and responsive to the diverse needs of all stakeholders within the context of the Ontario healthcare system.

### Data analysis

We will use a modified codebook thematic analysis aided by NVivo software. This method, guided by Braun and Clarke’s stages [64], involves familiarizing oneself with data (reviewing the transcripts and/or audio files numerous times), coding, and creating a comprehensive coding scheme. First, we will immerse ourselves in the data by repeatedly reviewing the transcripts and/or audio files to gain a deep understanding of the content. This familiarization process is crucial for developing an intuitive grasp of the data’s nuances. We will undertake both inductive and deductive coding to develop a detailed coding scheme. Inductive coding involves identifying patterns and themes directly from the data without any preconceived categories, allowing the data to guide the formation of codes based on observed content. In contrast, deductive coding uses existing theories or frameworks, such as the RACI framework and the Nine Pillars of Integration, to structure our coding. This ensures our analysis is anchored in established concepts. Creating a comprehensive coding scheme involves several steps. We will review and refine the initial codes, compare these codes against the RACI and the Nine Pillars of Integrated Care framework, and incorporate feedback from the research team to ensure the scheme covers all relevant aspects of the data. This collaborative process ensures a robust and exhaustive coding scheme.

As we progress, we will group similar codes into broader categories to identify key themes and sub-themes. This synthesis and categorization process helps pinpoint major patterns within the data, facilitating a clearer understanding of the overarching themes. During the thematic analysis phase, we will focus on identifying innovative practices, challenges, and outcomes of integrated models. Innovative practices highlight new and effective methods for integration, while challenges refer to common difficulties and obstacles faced. Outcomes encompass the results and impacts of these integrated initiatives. We will compare our findings with existing literature to highlight similarities between our themes and previously identified initiatives. This comparative analysis helps identify elements of governance models that can be applied and refines our themes to ensure they are comprehensive and well-supported by both the data and existing research. The titles for our themes will be crafted to reflect insights directly derived from the data, concepts from the RACI framework [44] and the Nine Pillars of Integrated Care [46], and findings from the literature review. This ensures the themes are both data-driven and theoretically grounded. NVivo software will assist us by facilitating the coding process, organizing and managing the data effectively, and supporting the synthesis and analysis of our qualitative data. By following this structured approach, we aim to provide a detailed understanding of the roles and responsibilities within best practice integrated care innovations involving PC practices and community pharmacies.

The investigative team will review this list of codes, compare it to the RACI framework and the Nine Pillars of Integrated Care framework and provide feedback to create an exhaustive coding scheme that we will apply to all data. The coding process will involve multiple coders, each working individually initially. Subsequently, they will convene to compare and collaborate on their respective codes. As we synthesize the data, we will combine similar codes into larger categories [64]. While conducting thematic analysis, we will draw upon the innovative practices, challenges, and outcomes observed in integrated programs and innovations found in the literature. Comparative analysis with literature-driven initiatives will help identify applicable governance model elements, shaping the data-derived themes. Titles will be crafted to reflect both the data-driven insights, the RACI framework, the Nine Pillars of Integration and the ideas identified in the literature. We will also draw on insights from governance [65] and health policy [66] literatures to examine power dynamics within integrated models. This perspective will allow us to ask more focused questions of the data, such as: Whose interests are being prioritized in decision-making? How is authority distributed among pharmacists, FPs and NPs stakeholders? What are potential sources of conflict and mechanisms for resolution? Given the potential for scope of practice reform to reshape professional boundaries and challenge existing hierarchies, we will attend closely to the form and structure of relationships enabled within this emergent governance system.

## Discussion

Community pharmacists play a crucial role in healthcare systems worldwide, offering diverse services and often serving as the most accessible healthcare providers within communities. The recent expansion of pharmacists’ scope of practice in Ontario, granting them prescribing authority for common ailments, reflects a transformative shift in PC in the Ontario health care system [9,67,68]. This expansion aligns with broader trends seen in other Canadian provinces and countries, indicating a global recognition of the value pharmacists bring to healthcare delivery. Governance mechanisms are essential for defining roles, responsibilities, and accountability among stakeholders, ensuring alignment with legal and ethical standards, and optimizing resource utilization.

This protocol outlines an exploratory study aimed at gathering foundational knowledge for developing an effective governance structure supporting integrated service delivery within the Ontario healthcare system. By conducting a comprehensive literature review and engaging key stakeholders through interviews, the study seeks to identify best practices, challenges, and priorities for governance. The use of a multi-methods approach, informed by the RACI framework and the Nine Pillars of Integration, ensures a holistic understanding of governance needs and opportunities, guiding the development of tailored governance strategies.

Overall, the findings from this study have the potential to inform policy and practice, facilitating the establishment of governance models that promote seamless integration among community pharmacists and FPs and NPs. A key expected outcome is improved integrated care, or the embedding of pharmacy and FP and NP services within a unified delivery system. This is expected to yield numerous downstream benefits, including improved coordination between providers (as demonstrated by such indicators as the development of joint care plans and increased interdisciplinary meetings) [69], enhanced patient outcomes (e.g., management of chronic conditions, adherence to medication regimens, reduced medication errors, fewer hospitalizations) [70], improved workforce conditions and service accessibility (e.g., alleviating physician workload by assigning pharmacists basic clinical responsibilities) [71], and greater system efficiency and cost-effectiveness (e.g., by reducing duplication of services and streamlining medication management processes) [72].

Critically, integrated care through shared governance between community pharmacists and FPs and NPs can also be expected to foster a more equitable distribution of power and improve health, economic, and humanistic outcomes across healthcare sectors, providers, and patients. Community pharmacists, whose role has traditionally been restricted to dispensing medications, may gain greater autonomy and influence in clinical decision-making through collaboration [73,74]. By assigning pharmacists basic clinical responsibilities, FP and NP workloads can be alleviated, supporting provider morale and retention of the workforce [71,75]. At the patient level, this can translate to increased attachment and service accessibility, particularly for historically underserved populations, including racialized communities, gender and sexual minorities, recent immigrants, individuals with low income, and those in rural areas [76]. Nevertheless, it is imperative to remain mindful of potential unintended consequences and consider strategies to mitigate risks. For example, the shifting of service delivery responsibilities to private, for-profit entities may introduce perverse incentives that undermine service quality and accessibility, which could disproportionately impact equity-deserving groups [77].

To the best of our knowledge, this study is the first of its kind. Integrated governance models hold the promise of becoming foundational elements in healthcare policy and practice. Ultimately, these models can enhance patient-centred care delivery, improve healthcare outcomes, and contribute to the overarching goal of enhancing the quality and accessibility of healthcare services.

## Conclusion

The outlined protocol signifies a pivotal advancement in fostering governance within the healthcare system to enhance patient-centred care. Community pharmacists have expanded roles following recent legislative changes, necessitating effective governance structures to ensure seamless integration with FPs and NPs. Through a comprehensive approach informed by the RACI framework and the Nine Pillars of Integration, this study seeks to gather foundational knowledge for developing such governance models. By conducting a thorough literature review and engaging stakeholders via interviews, the study aims to identify best practices, challenges, and priorities for a governance model that enables integration. These efforts not only pave the way for tailored strategies aligned with legal and ethical standards but also hold the potential to transform healthcare policy and practice. The envisioned governance models aim to enhance patient-centred care delivery, improve healthcare outcomes, and contribute to the overall enhancement of healthcare services in Ontario. The proposed model also has the potential to empower frontline providers, particularly community pharmacists, by expanding their role in decision-making and care coordination. By fostering clearer accountability and collaboration, the model could enable pharmacists to contribute more actively to patient care, ensuring that diverse healthcare providers have a voice in shaping healthcare delivery. Moreover, by facilitating greater collaboration across professions, the model can help break down disciplinary silos, fostering a more cohesive and team-based approach to primary care. This pioneering endeavour lays the groundwork for innovative governance approaches that could serve as a blueprint for integrated healthcare systems globally, positioning Ontario at the forefront of healthcare innovation and excellence.

## Supporting information

S1 Appendix PRISMA-P Checklist. (DOCX)

S2 Appendix Ovid Medline Search Strategy. (DOCX)

## Abbreviations

FP: family physician
IFIC: International Foundation for Integrated Care
NP: nurse practitioner
OHT: Ontario Health Team
PC: primary care
RACI: responsible, accountable, consulted, informed
