# Supplementary material for: Informing a governance model for integration of community pharmacists and family physicians and nurse practitioner-led practices and teams within Ontario Health Teams: A protocol
Source: PLoS One. 2025 Jun 17;20(6):e0325270. doi: 10.1371/journal.pone.0325270 (PMC12173222; doi:10.1371/journal.pone.0325270)
Supplement: S2 Appendix — (DOCX) [file pone.0325270.s002.docx]

**Ovid MEDLINE SEARCH STRATEGY:**

Epub Ahead of Print, In-Process & Other Non-Indexed Citations, Ovid MEDLINE® Daily and Ovid MEDLINE® <1946-Present>

1. exp General Practice/ or General Practice.kf,tw. 99413
2. exp Family Practice/ or Family Practice.kf,tw. 71155
3. exp Nursing, Team/ or Nursing, Team.kf,tw. 3807
4. exp Patient Care Team/ or Patient Care Team.kf,tw. 74004
5. exp Primary Health Care/ or Primary Health Care.kf,tw. 216633
6. exp Comprehensive Health Care/ or Comprehensive Health Care.kf,tw.
7. exp "Delivery of Health Care"/ or (delivery adj3 health).kf,tw. or "Delivery of Health Care".kf,tw.
8. exp Preventive Medicine/ or Preventive Medicine.kf,tw.
9. exp Pharmacy Services/ or Community Pharmacy Service.kf,tw. or exp Pharmacists/ or Pharmacists.kf,tw. or pharmaceutical.kf,tw. or "Pharmacy" or "Pharmacists".kf,tw.or "Pharmacy Services".kf,tw.  150406
10. exp professional role/ or professional role.kf,tw. 91626
11. exp Intersectoral Collaboration/ or Intersectoral Collaboration.kf,tw. 3231
12. exp Cooperative Behavior/ or Cooperative Behavior.kf,tw. 47239
13. exp Interdisciplinary Communication/ or Interdisciplinary Communication.kf,tw.
14. exp Clinical Decision-Making/ or Clinical Decision-Making.kf,tw. 44386
15. exp "Attitude of Health Personnel"/ or "Attitude of Health Personnel".kf,tw. 172103
16. exp Group Processes/ or Group Processes.mp. 197231
17. exp Group Dynamics/ or Group Dynamics.mp. 2458
18. 1 or 2 or 3 or 4 or 5 or 6 or 7 or 8 1836898
19. 10 or 11 or 12 or 13 or 14 or 15 or 16 or 17 415223
20. 9 and 18 and 19 9843
21. limit 21 to yr="2013 -Current"**9843**
